# Three-Degree-of-Freedom Voice Coil Actuator Driven by a Four-Phase Current

**DOI:** 10.3390/s22186926

**Published:** 2022-09-13

**Authors:** Akira Heya, Katsuhiro Hirata

**Affiliations:** 1 Department of Mechanical Systems Engineering, Nagoya University, Furo-cho, Chikusa-ku, Nagoya 464-8603, Aichi, Japan; 2 Division of Materials and Manufacturing Science, Osaka University, 2-1 Yamadaoka, Suita 565-0871, Osaka, Japan

**Keywords:** actuator, multiple-degree-of-freedom actuator, voice coil actuator

## Abstract

Camera attitude control systems for robots require a compact structure and high responsiveness. However, due to the combination structure of several actuators, the camera attitude control system is large. To address this issue, this study proposes a three-degree-of-freedom (3DOF) voice coil actuator. A single actuator is used to generate 3DOF motion, which is driven by a four-phase current. This study also describes the basic structure and operating principle of the actuator and clarifies the torque characteristics using a three-dimensional (3D) finite element method (FEM). Furthermore, the dynamic modeling and control methods are presented. The FEM and dynamic simulation results reveal that the proposed actuator can be arbitrarily driven in 3DOF.

## 1. Introduction

Surrounding recognition is important for autonomous mobile systems such as robots, drones, and self-driving cars [1,2,3]. These systems gather visual information using a camera in an unknown environment, necessitating a high level of spatial awareness. However, image deterioration occurs due to its vibration. Furthermore, the size increases due to a multiple-degree-of-freedom (DOF) mechanism for changing the camera attitude. Various parallel link mechanisms have been developed to generate the multiple-DOF motion [4,5,6,7]; however, the mechanisms have some disadvantages, such as a large number of components, structural complexity, and narrow driving angle. To solve these problems, this study focuses on multiple-DOF actuators. They can generate multiple-DOF motions with a single actuator.

Various types of multiple-DOF actuators have been proposed: synchronous [8,9,10], stepping [11,12], and induction motor types [13]. However, these conventional actuators require a control device and structure of a large size to achieve a high torque density and continuous rotation. In the driving source for the camera attitude control system, a compact structure with a few components and high responsiveness is required. Moreover, it must be able to be driven using a simple control device. Thus, this study proposed a novel three-DOF voice coil actuator (3DOFVCA) driven by only a four-phase current, with the minimum possible configuration [14].

This study describes the structure, operating principle, and dynamics of the proposed 3DOFVCA. The torque characteristics are investigated using a three-dimensional finite element method (3D FEM). The FEM analysis results show that the 3DOFVCA can generate torque on the three-axis. Furthermore, the dynamic modeling and control methods are presented. The dynamic simulation results clarify that the proposed actuator can be controlled in 3DOF.

## 2. Three-Degree-of-Freedom Voice Coil Actuator

### 2.1. Basic Structure

**Figure 1:**
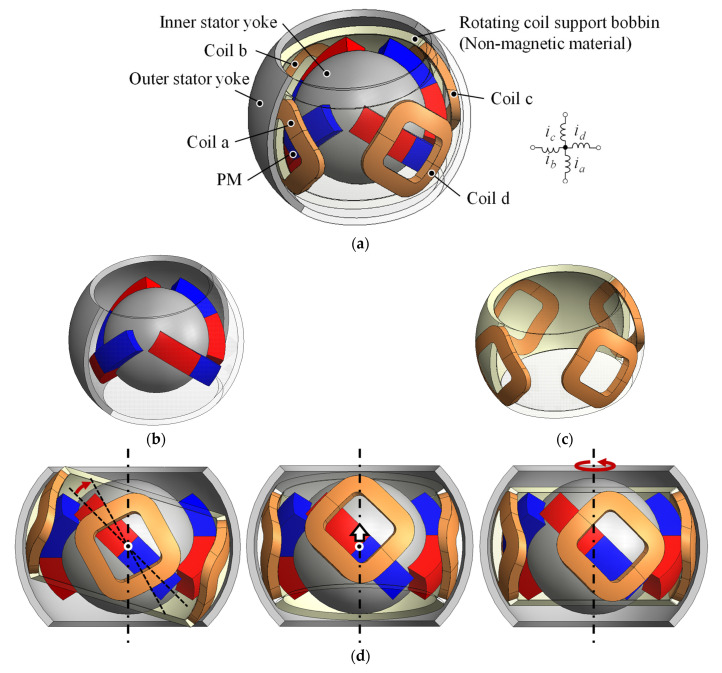
The 3DOFVCA driven by four-phase current: (**a**) entire view; (**b**) stator; (**c**) rotor; and (**d**) motion of the rotor. The half of the outer stator yoke and rotating coil support are translucent for visibility.

**Figure 2:**
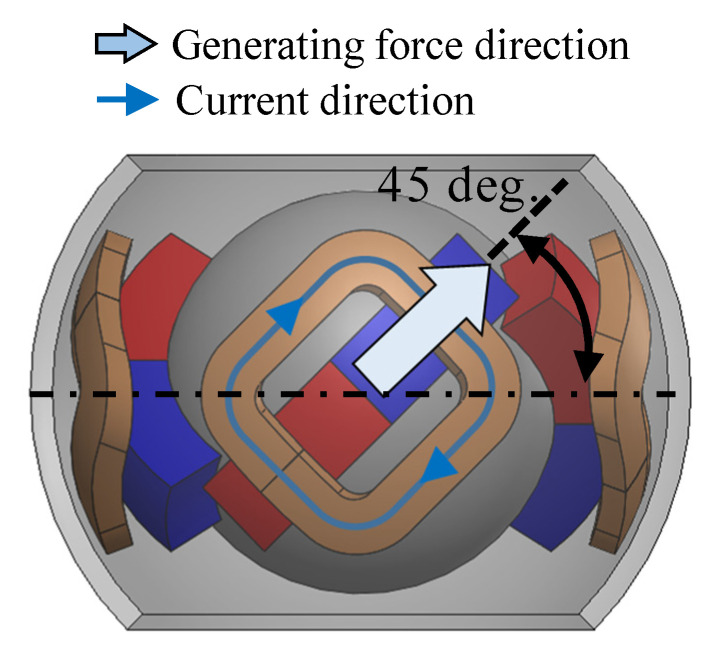
Side view of the 3DOFVCA when the rotor position is the origin.

Figure 1 and Figure 2 show the overview and side views of the proposed 3DOFVCA, respectively. Four coils and permanent magnets (PMs) tilted 45° were arranged at an interval of 90°. The PMs were arranged with alternating positive and negative 45° inclinations. This actuator includes a moving coil structure with four coils. The coils were coupled at a neutral point, and a spherical bearing mechanically supported the rotor. The stator was made up of spherical PMs and outer and inner yokes. The actuator is a surface permanent magnet structure and has no salient pole. The magnetic circuit is independent and symmetric for each of the four PMs. The proposed actuator can generate 3DOF motion by only one rotor with four coils, four magnets, two yokes, and one spherical bearing. Therefore, the advantage of the actuator is the integration of electromagnetic force generation mechanisms within a spherical volume.

**Figure 3:**
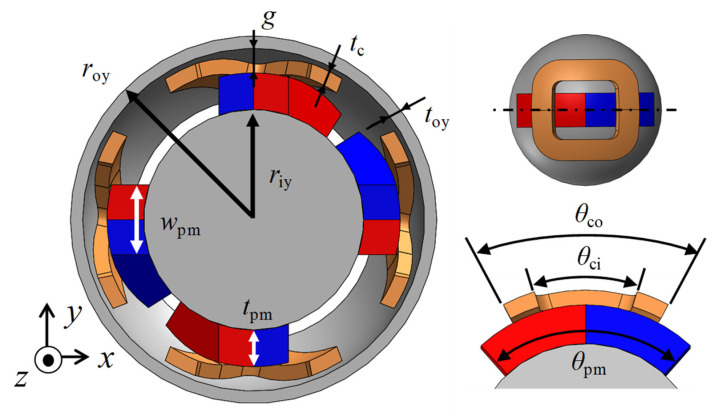
Design parameters.

The model was designed for principle verification. The outer diameter of the stator was 30 mm, and the number of turns was 40. Figure 3 and Table 1 show the detailed design parameters and the parameter values, respectively.

### 2.2. Operating Principle

**Figure 4:**
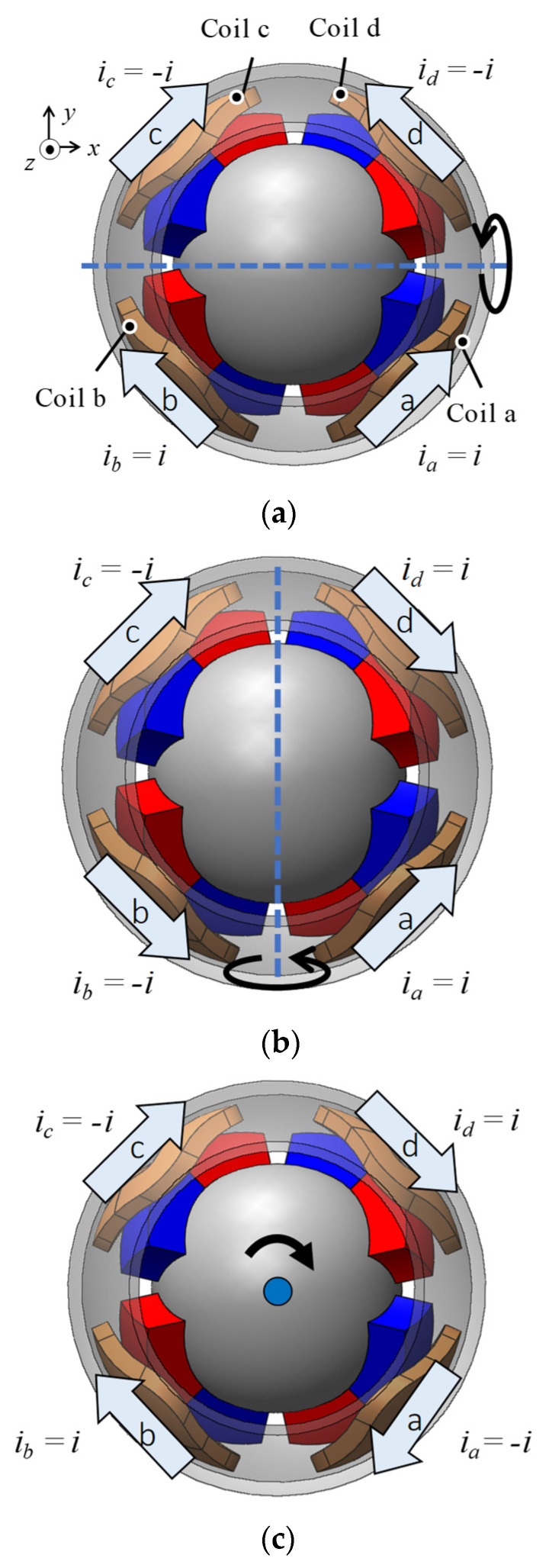
Operating principle of the 3DOFVCA: (**a**) X-axis; (**b**) Y-axis; and (**c**) Z-axis.

A magnetic attraction force was generated using one exciting coil, as shown in Figure 2. The 3DOF motion was achieved by adjusting the current direction in each coil (see Figure 4). Table 2 presents the torque generation patterns. The currents in each phase were applied in an equilibrium state of the current in an electric circuit. This means that the sum of the currents in each phase is zero at the neutral point. Due to its structural and magnetic symmetry, this current equilibrium condition is also established during rotation. This is because the magnetized direction of the PM alternates between N and S poles when viewed circumferentially. Therefore, the actuator was driven by a four-phase half-bridge circuit. The torque is generated because the flux linkage is changed by rotation.

## 3. Torque Characteristics

The torque characteristics of the proposed 3DOFVCA were investigated by employing the 3D FEM through a magnetic field analysis using a T-ω method (MAGNET, Siemens) [15,16]. The analysis validity was confirmed in reference [17] (see Appendix A). The analysis conditions and results of the basic model are shown, and the PM arrangement’s structural comparison is presented.

### 3.1. Analysis Conditions

**Figure 5:**
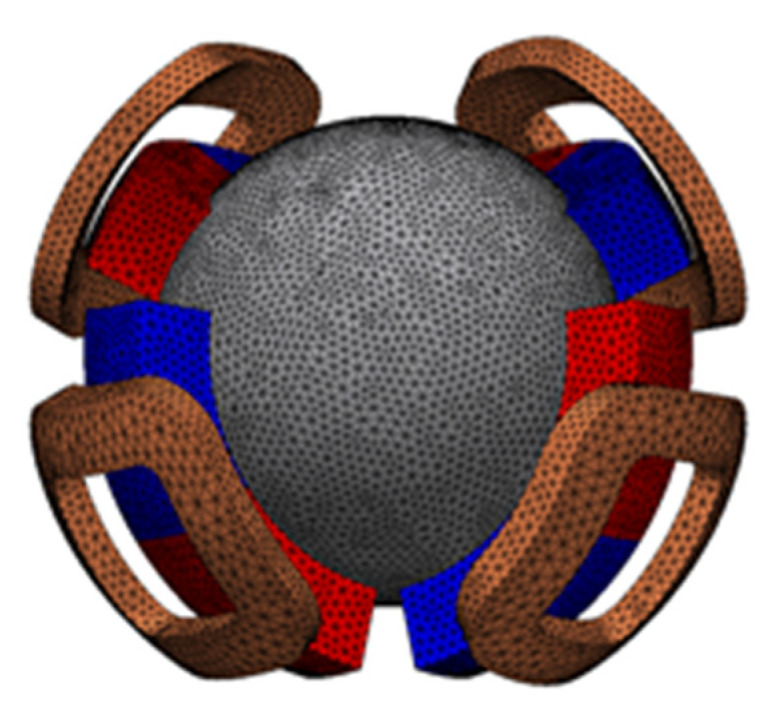
Three-dimensional mesh model excluding the air region and outer stator yoke.

Figure 5 shows the 3D mesh model of the basic model, excluding air. The number of elements was approximately 2,360,000. The residual magnetic flux density of the PM was 1.3 T, and the coercive force was 1.05 × 10^6^ A/m. The yokes were made of electromagnetic soft iron (SUY). The limit in the current was a current density of 20 A/mm^2^.

### 3.2. Results

**Figure 6:**
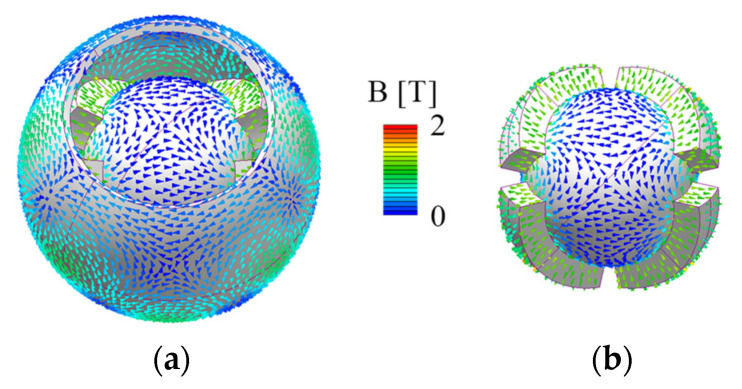
Magnetic flux density distribution of the 3DOFVCA: (**a**) whole view and (**b**) without outer yoke and coils.

**Figure 7:**
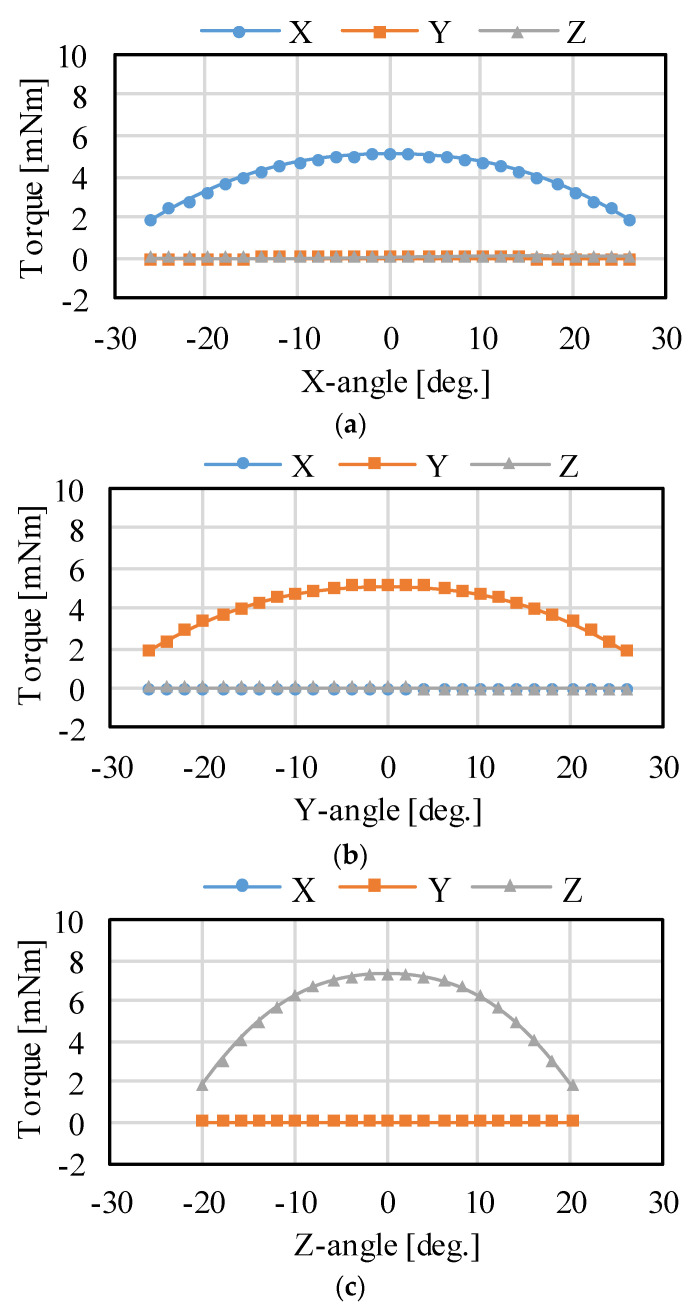
Analysis results of the static torque: (**a**) rotation around the X-axis; (**b**) rotation around the Y-axis; and (**c**) rotation around the Z-axis.

Figure 6 shows the magnetic flux density distribution. It was confirmed that the magnetic circuit between the PM and yokes is symmetric. The torque characteristics were calculated when a current was applied to generate the torque around each axis. Only the torque around the driving axis is generated, as shown in Figure 7. The torque fluctuation during rotation is caused by the approach of the pole center of the coils and PMs. The facing area between the coil and PM is small in rotation around the Z-axis compared to the rotation around the X- and Y-axis. Therefore, the driving range around the Z-axis is narrow.

### 3.3. Structural Comparison

**Figure 8:**
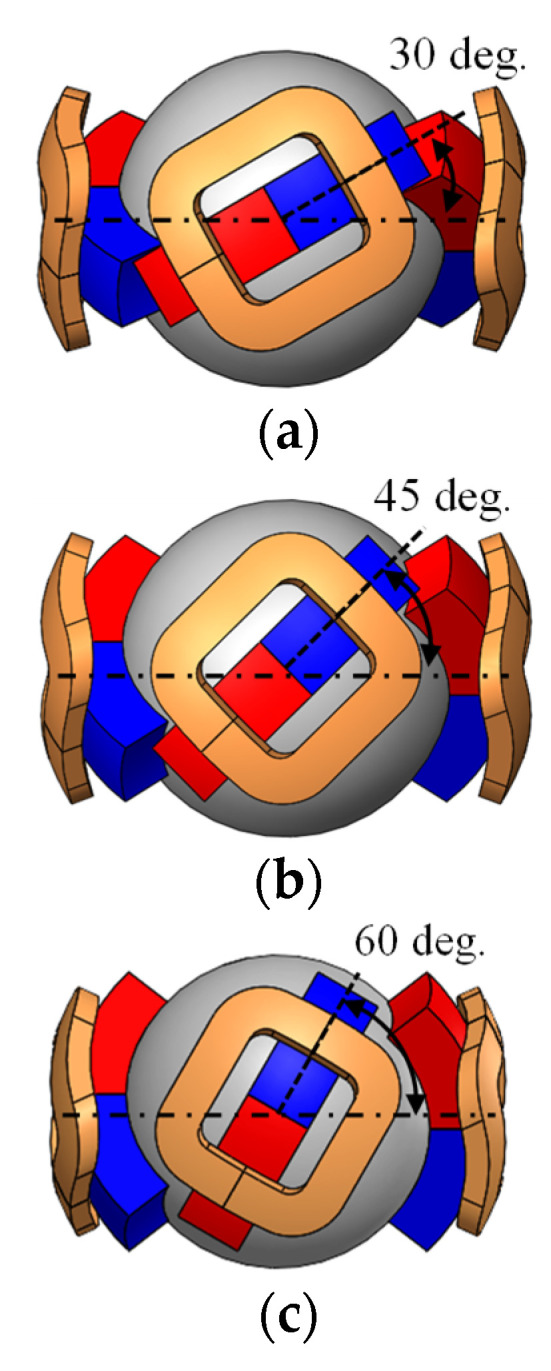
Placement angle of the PM: (**a**) 30°; (**b**) 45°; and (**c**) 60°.

**Figure 9:**
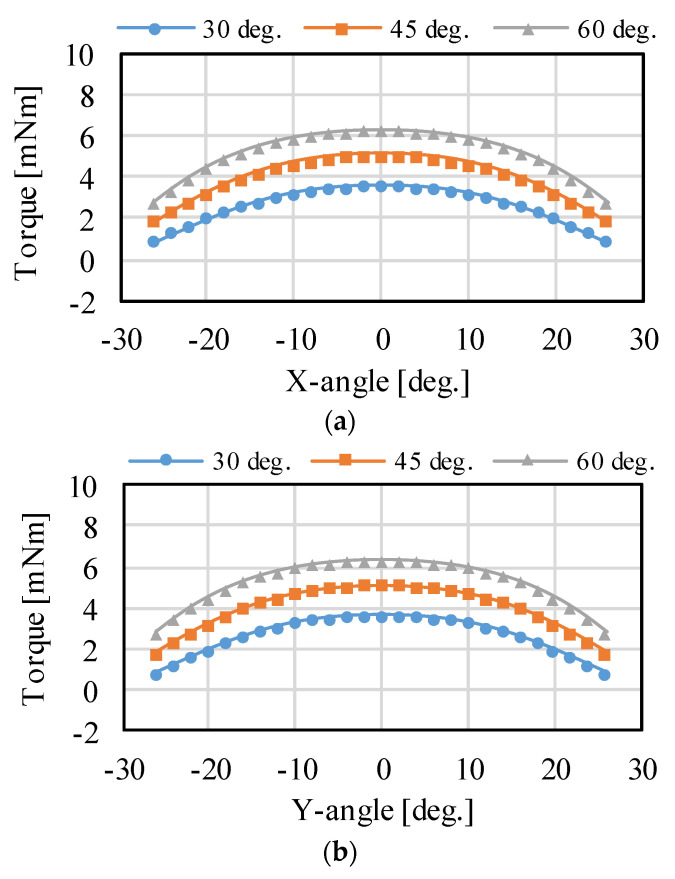

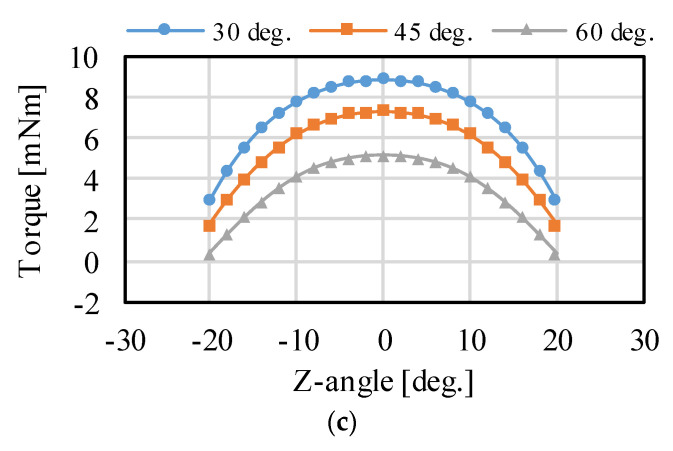
Torque characteristics when the placement angles of the PMs are changed: (**a**) rotation around the X-axis; (**b**) rotation around the Y-axis; and (**c**) rotation around the Z-axis.

The effect of the changing PM placement angle is verified as shown in Figure 8 and Figure 9, which present the analyzed results. The torque around the X- and Y-axis becomes larger by increasing the placement angle. However, the torque around the Z-axis becomes smaller. This is because the force vector direction in each coil changes. The driving range around the X- and Y-axis expands, whereas that of the Z-axis narrows. The torque can be distributed for each axis by adjusting the PM placement angle, although there is a trade-off in the maximum torque and driving range. Thus, it is necessary to design according to the application.

## 4. Modeling and Control Method

This section describes the dynamic modeling and control method. The dynamics of the proposed 3DOFVCA are expressed as electrical and mechanical responses. Furthermore, the circuit and motion equations are derived. The control method for controlling 3DOF motion by a four-phase current is presented.

### 4.1. Dynamic Modeling

First, the electrical dynamics was considered. The circuit equation is as follows:

(1) $$\left[\begin{array}{c} v_{a}\\ v_{b}\\ v_{c}\\ v_{d} \end{array}\right]=\left[\begin{array}{cccc} R_{a} & 0 & 0 & 0\\ 0 & R_{b} & 0 & 0\\ 0 & 0 & R_{c} & 0\\ 0 & 0 & 0 & R_{d} \end{array}\right]\left[\begin{array}{c} i_{a}\\ i_{b}\\ i_{c}\\ i_{d} \end{array}\right]+\frac{d}{dt}\left\{\left[\begin{array}{cccc} L_{aa} & M_{ab} & M_{ac} & M_{ad}\\ M_{ba} & L_{bb} & M_{bc} & M_{bd}\\ M_{ca} & M_{cb} & L_{cc} & M_{cd}\\ M_{da} & M_{db} & M_{dc} & L_{dd} \end{array}\right]\left[\begin{array}{c} i_{a}\\ i_{b}\\ i_{c}\\ i_{d} \end{array}\right]\right\}+\left[\begin{array}{c} e_{a}\\ e_{b}\\ e_{c}\\ e_{d} \end{array}\right]$$

where *v* is the voltage, *I* is the current, *R* is the coil’s resistance, *L* is the self-inductance, *M* is the mutual inductance, and *e* is the back-EMF. In this actuator, the inductance can be regarded as constant because of the narrow rotational range. From the symmetry of the magnetic circuit, the mutual inductances can be considered the same:

(2) $$R_{a}=R_{b}=R_{c}=R_{d}\equiv R,\;\;L_{aa}=L_{bb}=L_{cc}=L_{dd}$$

(3) $$M_{ab}=M_{ac}=M_{ad}=M_{bc}=M_{bd}=M_{cd}\equiv M$$

In the equilibrium state of the current, the following terms are given:

(4) $$Mi_{b}+Mi_{c}+Mi_{d}=-Mi_{a},\;Mi_{a}+Mi_{c}+Mi_{d}=-Mi_{b}$$

(5) $$Mi_{a}+Mi_{b}+Mi_{d}=-Mi_{c},\;Mi_{a}+Mi_{b}+Mi_{c}=-Mi_{d}$$

**Figure 10:**
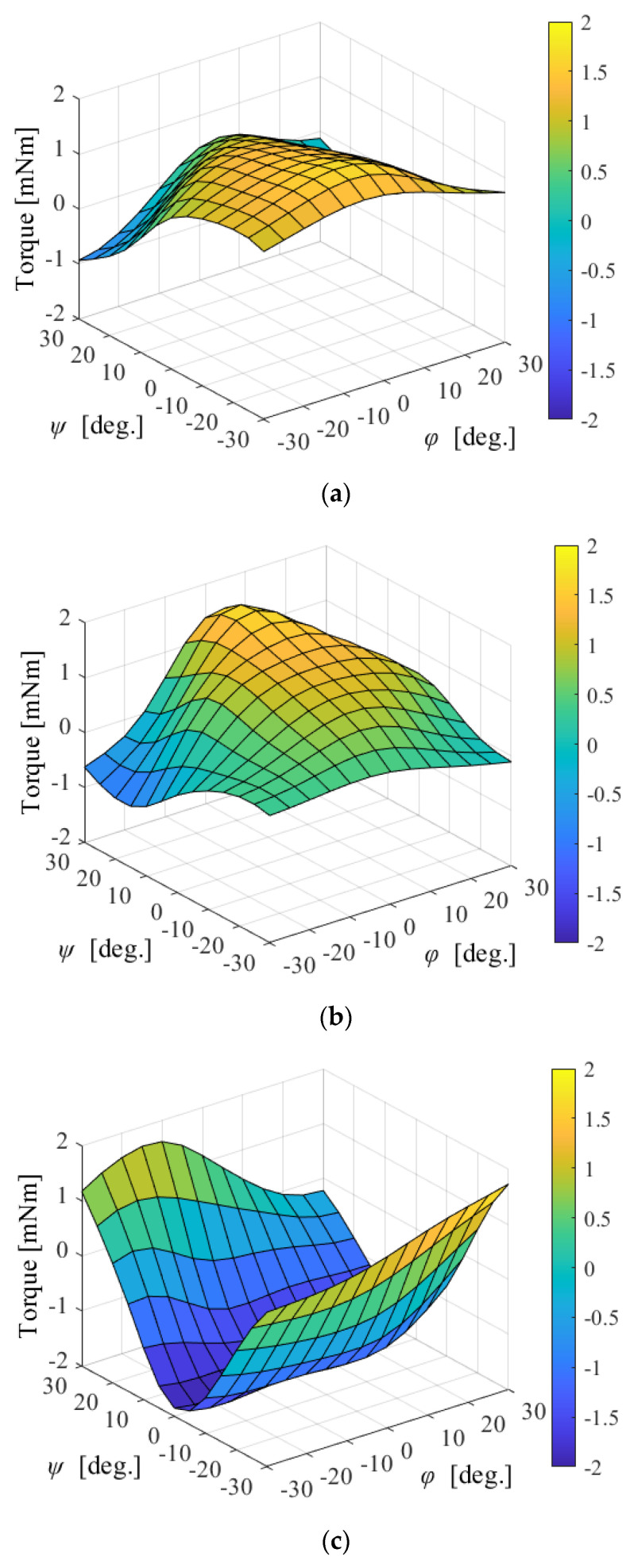
Torque constant map in *a*-phase: (**a**) rotation around the X-axis; (**b**) rotation around the Y-axis; and (**c**) rotation around the Z-axis.

From Equations (2)–(5), the circuit equation can be converted as follows:

(6) $$\left[\begin{array}{c} v_{a}\\ v_{b}\\ v_{c}\\ v_{d} \end{array}\right]=\left[\begin{array}{cccc} R+\rho L & 0 & 0 & 0\\ 0 & R+\rho L & 0 & 0\\ 0 & 0 & R+\rho L & 0\\ 0 & 0 & 0 & R+\rho L \end{array}\right]\left[\begin{array}{c} i_{a}\\ i_{b}\\ i_{c}\\ i_{d} \end{array}\right]+\left[\begin{array}{c} e_{a}\\ e_{b}\\ e_{c}\\ e_{d} \end{array}\right]$$

where *ρ* = d/dt and *L* = *L*_aa_ – *M*. Next, the conversion between the electrical and mechanical energies is expressed as follows:

(7) $$\tau =K_{T}(\varphi ,\psi )i$$

(8) $$K_{T}(\alpha ,\beta ,\gamma )=\left[\begin{array}{cccc} K_{Txa}(\varphi ,\psi ) & K_{Txb}(\varphi ,\psi ) & K_{Txc}(\varphi ,\psi ) & K_{Txd}(\varphi ,\psi )\\ K_{Tya}(\varphi ,\psi ) & K_{Tyb}(\varphi ,\psi ) & K_{Tyc}(\varphi ,\psi ) & K_{Tyd}(\varphi ,\psi )\\ K_{Tza}(\varphi ,\psi ) & K_{Tzb}(\varphi ,\psi ) & K_{Tzc}(\varphi ,\psi ) & K_{Tzd}(\varphi ,\psi ) \end{array}\right]\;\tau ={\left[\tau_{x}\;\tau_{y}\;\tau_{z}\right]}^{T},\;i={\left[i_{a}\;i_{b}\;i_{c}\;i_{d}\right]}^{T}$$

where ***τ*** is the torque and ***K****_T_* is the torque constant. The torque constant was changed by the rotation. Therefore, the coil position vectors in each phase are defined using *φ* and *ψ*, where *φ* is the longitude of the coil position vector and *ψ* is the latitude. The torque was calculated as the total torque in each coil. For example, Figure 10 shows the torque constant maps of the *a*-phase. The attitude is expressed by the XYZ-Euler angle (*α*, *β*, *γ*). The rotation matrix is given as follows:

(9) $$R_{tr}=\left[\begin{array}{ccc} c\beta c\gamma & -c\beta s\gamma & s\beta \\ c\gamma s\alpha s\beta +c\alpha s\gamma & c\alpha c\gamma -s\alpha s\beta s\gamma & -c\beta s\alpha \\ -c\alpha c\gamma s\beta +s\alpha s\gamma & c\gamma s\alpha +c\alpha s\beta s\gamma & c\alpha c\beta \end{array}\right]$$

where c and s represent cosine and sine, respectively.

The mechanical dynamics was obtained using the Lagrange equation of motion as follows:

(10) $$\frac{d}{dt}\frac{\partial L_{q}}{\partial \dot{q}}-\frac{\partial L_{q}}{\partial q}=\tau$$

where *q* is the generalized coordinate, and ***L****_q_* is the Lagrangian. ***L****_q_* is defined as follows:

(11) $$L_{q}(q,\dot{q})=K(q,\dot{q})-P(q)$$

where ***K*** is the kinetic energy and ***P*** is the potential energy. ***K*** is defined as follows:

(12) $$K(q,\dot{q})=\frac{1}{2}\omega^{T}R_{tr}IR_{tr}^{T}\omega$$

where ***ω*** is the angular velocity and ***I*** is the tensor of inertia. ***ω*** is defined as follows:

(13) $$\omega =\left[\begin{array}{ccc} 1 & 0 & s\beta \\ 0 & c\alpha & s\alpha c\beta \\ 0 & s\alpha & c\alpha c\beta \end{array}\right]\left[\begin{array}{c} \dot{\alpha }\\ \dot{\beta }\\ \dot{\gamma } \end{array}\right]$$

***I*** is defined as follows:

(14) $$I=diag[\begin{array}{ccc} I_{xx} & I_{yy} & I_{zz} \end{array}]$$

**Figure 11:**
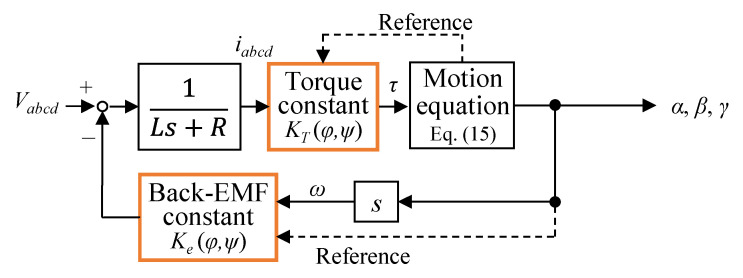
Block diagram of the 3DOFVCA.

The potential energy was assumed to be zero because the center mass of the rotor was located at the origin.

(15) $$H\ddot{q}+C\dot{q}=\tau$$

(16) $$H=\left[\begin{array}{ccc} I_{1}c^{2}\beta +I_{3}s^{2}\beta & 0 & I_{3}s\beta \\ 0 & I_{1} & 0\\ I_{3}s\beta & 0 & I_{3} \end{array}\right]$$

(17) $$C=\left[\begin{array}{ccc} 2\dot{\beta }(I_{3}-I_{1})s\beta c\beta & \dot{\gamma }I_{3}c\beta & 0\\ \dot{\alpha }(I_{1}-I_{3})s\beta c\beta & 0 & -\dot{\alpha }I_{3}c\beta \\ \dot{\beta }I_{3}c\beta & 0 & 0 \end{array}\right]$$

where ***q*** = [*α β γ*]*^T^*, *I*_1_ = *I_xx_* = *I_yy_*, and *I*_3_ = *I_zz_.* From Equations (6), (7) and (15), Figure 11 shows the 3DOFVCA model. In this model, the input was the voltage, and the output was the attitude. The torque constant was obtained from the results of the 3D FEM analysis. The back-EMF constant was assumed to be the same as the torque constant.

### 4.2. Control Method

**Figure 12:**
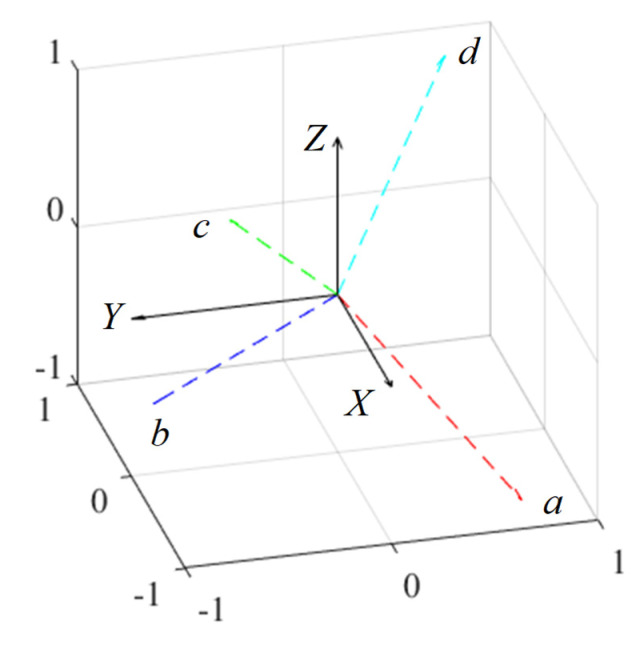
Coordinate system with the generated force vector.

**Figure 13:**
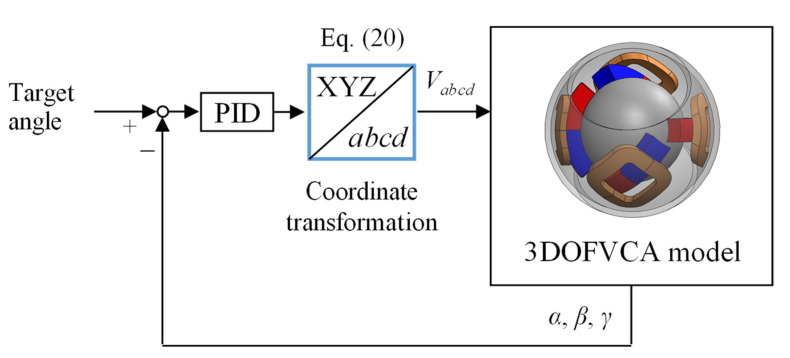
Attitude control system.

The coordinate transformation between *a*–*b*–*c*–*d* and *α*–*β*–*γ* coordinates are required to control the attitude of the actuator. Figure 12 shows the generated force vectors in each coil. The coordinate transformation matrix can be expressed as follows:

(18) $$\left[\begin{array}{c} f_{X}\\ f_{Y}\\ f_{Z} \end{array}\right]=T\left[\begin{array}{c} f_{a}\\ f_{b}\\ f_{c}\\ f_{d} \end{array}\right]$$

(19) $$\begin{array}{l} T=\left[\begin{array}{cccc} s\varphi c\psi & s(\varphi +\frac{1}{2}\pi )c(-\psi ) & s(\varphi +\pi )c\psi & s(\varphi +\frac{3}{2}\pi )c\psi \\ c\varphi c\psi & c(\varphi +\frac{1}{2}\pi )c(-\psi ) & c(\varphi +\pi )c\psi & c(\varphi +\frac{3}{2}\pi )c\psi \\ s(-\psi ) & s\psi & s(-\psi ) & s\psi \end{array}\right]\\ =\frac{1}{2}\left[\begin{array}{cccc} 1 & 1 & -1 & -1\\ 1 & -1 & -1 & 1\\ -\sqrt{2} & \sqrt{2} & -\sqrt{2} & \sqrt{2} \end{array}\right] \end{array}$$

where *f* is the physical quantity such as a voltage and current. The inverse transformation can be expressed as follows:

(20) $$\left[\begin{array}{c} f_{a}\\ f_{b}\\ f_{c}\\ f_{d} \end{array}\right]=T^{+}\left[\begin{array}{c} f_{X}\\ f_{Y}\\ f_{Z} \end{array}\right]$$

where ***T***^+^ is the pseudoinverse matrix. Figure 13 shows the attitude control system. A feedback control system using a PID controller can be used to control the attitude.

## 5. Dynamic Simulation

A dynamic simulation was conducted to verify the effectiveness of the control method. The time step for the simulation was set to 0.1 ms because the results did not change when the shorter time step was set. Table 3 presents the actuator parameters. The gain *k_p_*, *k_i_*, and *k_d_* were 1, 0.1, and 0.001, respectively. These values were designed by trial and error. The gains were set for stable convergence without overshoot and steady-state deviation. The time response of the system was calculated using the circuit equation Equation (1), torque generation equation Equation (7), and motion equation Equation (15). The variation of the torque constant and back-EMF constant were calculated as Lookup tables obtained through 3D FEM analysis. In addition, Simulink was used for a numerical calculation using a Runge–Kutta solver.

**Figure 14:**
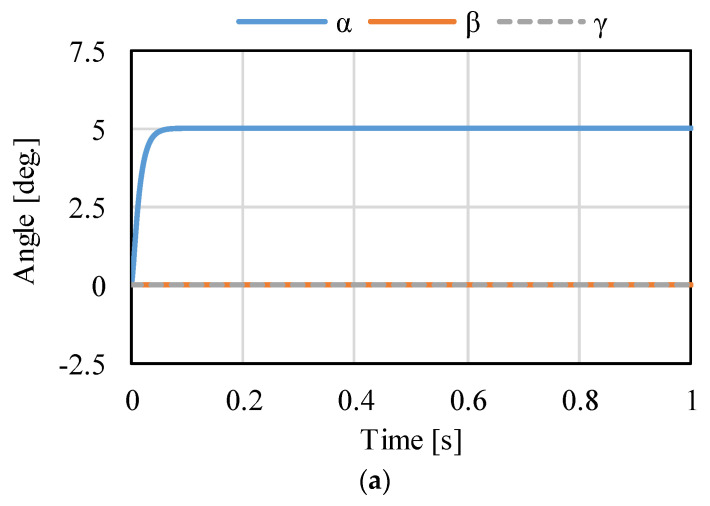

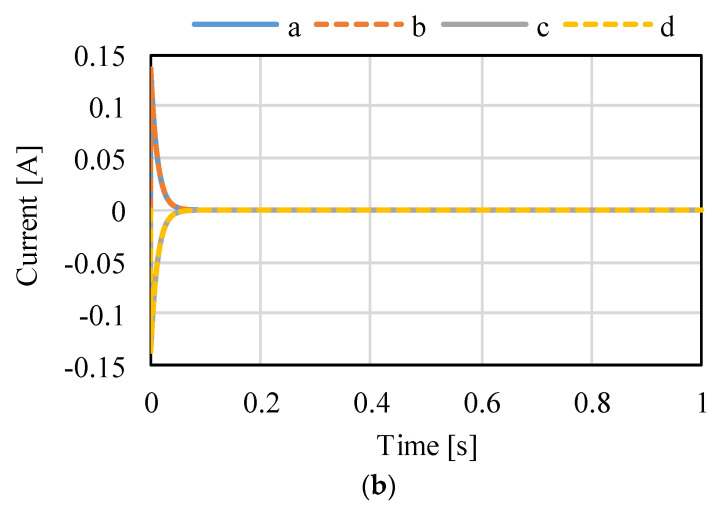
Calculated results when the target is the rotation in *α*: (**a**) attitude and (**b**) current.

**Figure 15:**
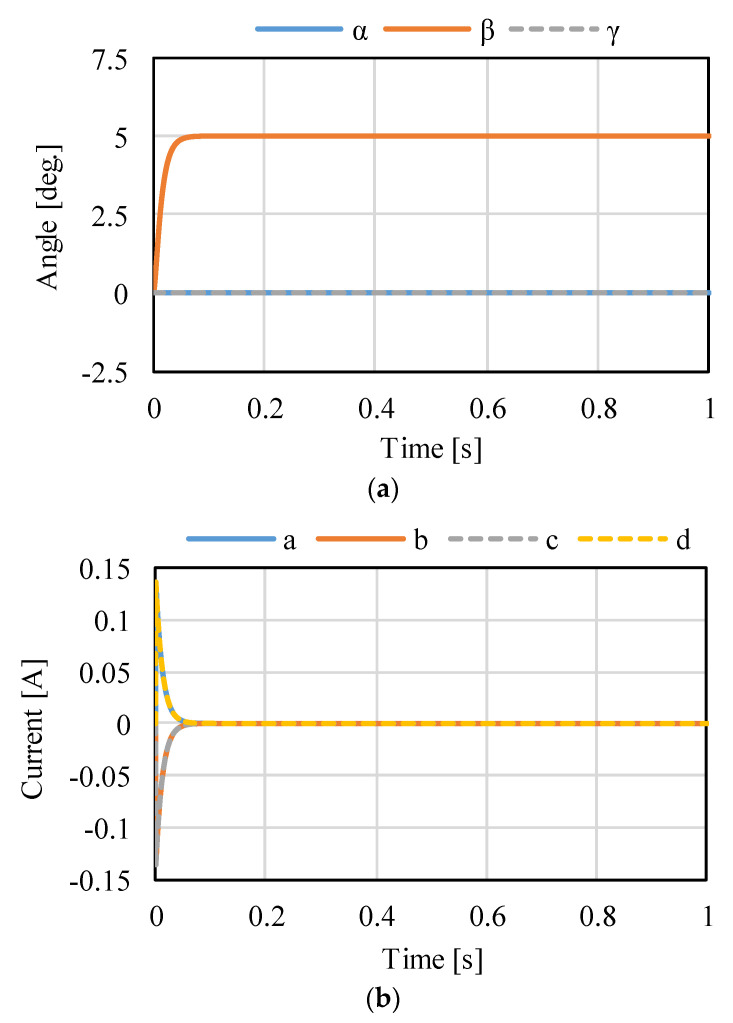
Calculated results when the target is the rotation in *β*: (**a**) attitude and (**b**) current.

**Figure 16:**
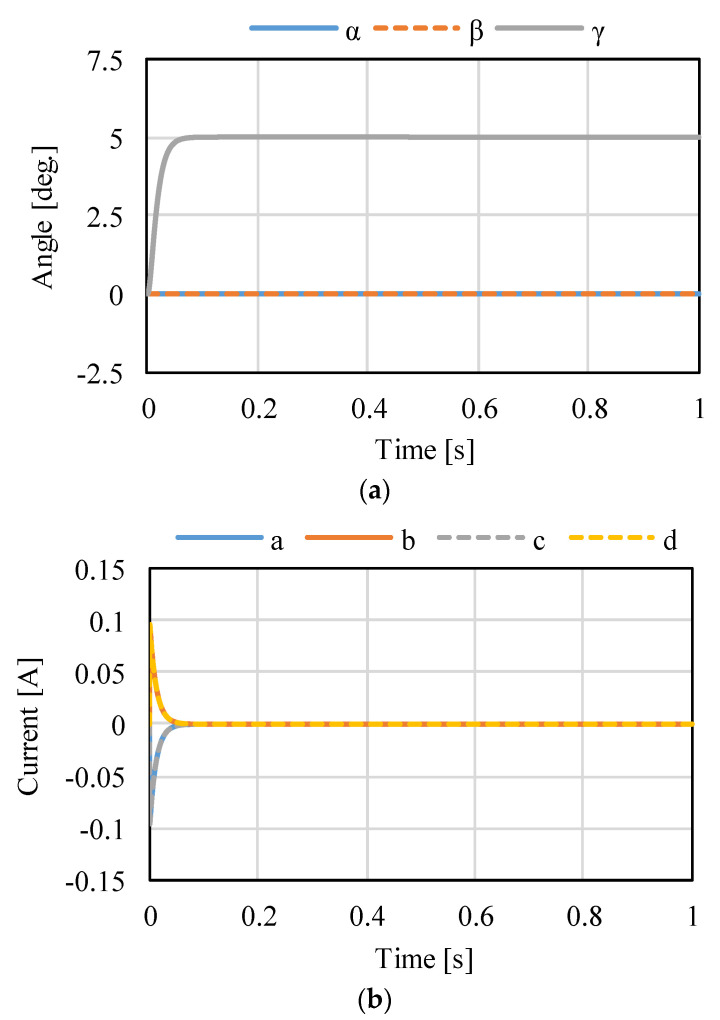
Calculated results when the target is the rotation in *γ*: (**a**) attitude and (**b**) current.

**Figure 17:**
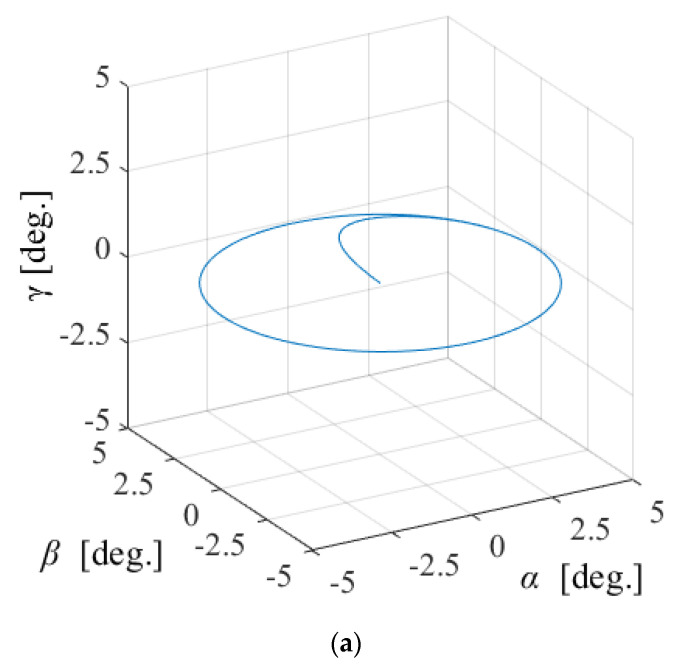

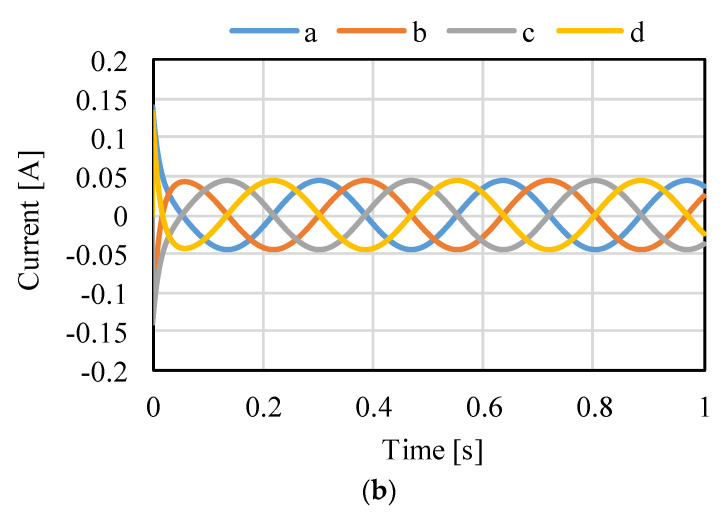
Circular motion in *α*-*β*: (**a**) attitude and (**b**) current.

Figure 14, Figure 15 and Figure 16 shows the control results for *α*-, *β*-, and *γ*-rotation. The target values were 5° in each simulation. The rotor was set to the target attitude, and there was a response delay in the transient state. The steady-state deviations were negligible in each direction. Figure 17 shows the results of the circular motion tracking. The rotor rotates along a circle, and the total currents between each phase are zero. Therefore, the equilibrium state was obtained. From these results, the actuator can be controlled using the proposed method. In this study, the target accuracy was set at 0.1° or less. In the actual system, friction compensation and identification are important because the accuracy decreases due to friction.

In an actual system, the dimensional specifications and actuator parameters may be slightly different. First, it is described when the dimensional parameters listed in Table 1 are different between the analysis and experiment due to the machining and assembly errors. The outer and inner yoke’s dimensional error changes the air gap length. When the gap increases due to the errors, the torque decreases by reduction of the magnetic resistance. However, the yoke made by a soft magnetic material has a smaller dimensional error than a permanent magnet, so the effect is small. On the other hand, a permanent magnet has a larger dimensional error than yokes. Therefore, the torque differs slightly between experiment and analysis due to minute changes in size; however, the proposed control method is effective in this case as well. As for the coil dimensions, if the coil can be manufactured with the designed number of turns, the effect on the torque is small. To increase the torque density, the winding space factor must be improved to increase the magnetomotive force. Next, the parameter change listed in Table 3 is described. The inertia error affects the mechanical responsiveness; however, the effect is small because the machining error is small. The change of coil resistance and inductance affects the electrical responsiveness. The viscous friction coefficient determines the damping characteristics and affects acceleration performance.

## 6. Conclusions

This study proposed a novel 3DOFVCA driven by a four-phase current. The torque characteristics were clarified by employing the 3D FEM. The results showed that the actuator can generate the torque on the three-axis in the rotational range of ±20°. The actuator is 30 mm in diameter and generated a maximum torque of about 7 mNm. The limitation of this actuator is that it cannot rotate infinitely. A dynamic modeling method was proposed using the circuit, energy conversion, and motion equations. The dynamic simulation results confirmed the validity of the proposed attitude control method using the coordinate transformation between the four-phase voltage and Euler angle. 

## Figures and Tables

**Table 1:** Design parameter values.

Symbol	Description	Value
*r*_oy_	Outer radius of the outer yoke	15.0 mm
*r*_iy_	Outer radius of the inner yoke	9.0 mm
*t*_oy_	Thickness of the outer yoke	1.0 mm
*t*_c_	Thickness of the coil	1.2 mm
*t*_pm_	Thickness of the PM	3.0 mm
*w*_pm_	Width of the PM	4.0 mm
*θ*_pm_	Angle of the PM	85.0°
*θ*_ci_	Inner angle of the coil	34.0°
*θ*_co_	Outer angle of the coil	56.0°
*g*	Air gap length	2.0 mm
	Opening angle of the outer yoke	90.0°

**Table 2:** Current pattern for the torque generation.

Torque	Coil a	Coil b	Coil c	Coil d
*τ_x_*	1	1	−1	−1
*τ_y_*	1	−1	−1	1
*τ_z_*	−1	1	−1	1

**Table 3:** Actuator parameter values.

Variable	Description	Value
*I_xx_* (=*I_yy_*)	Inertia of moment [10^−7^ × kgm^2^]	3.6
*I_zz_*		6.0
*R*	Resistance [Ω]	0.31
*L*	Inductance [mH]	0.0349
*M*	Mutual inductance [mH]	0
*D*	Viscous friction coefficient [Nms/rad]	0.0001

**Figure A1:**
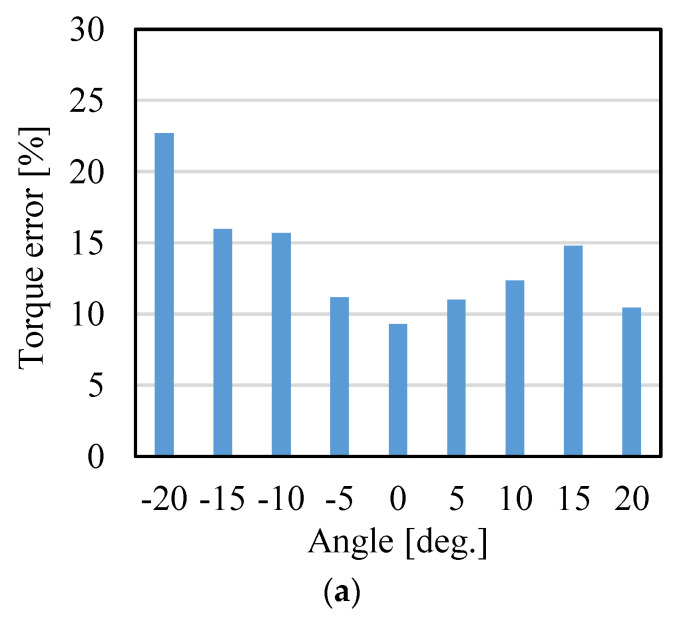

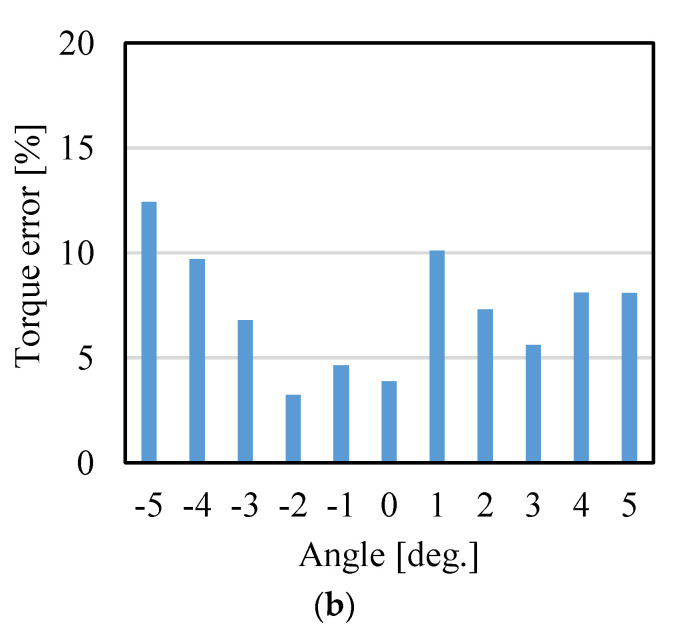
Validity of torque analysis: (**a**) X-axis and (**b**) Z-axis.

## Appendix A

In this study, a 3D FEM analysis software (MAGNET, Siemens) was used to evaluate the torque characteristics of a proposed actuator. In [17], the qualitative validity of the analysis was clarified by comparison of analysis and experiment, as shown in Figure A1.
